# Plaque‐Targeted Rapamycin Spherical Nucleic Acids for Synergistic Atherosclerosis Treatment

**DOI:** 10.1002/advs.202105875

**Published:** 2022-03-28

**Authors:** Yuanyuan Guo, Jingcan Qin, Qianqian Zhao, Jiapei Yang, Xiaoer Wei, Yu Huang, Miao Xie, Chuan Zhang, Yuehua Li

**Affiliations:** ^1^ Department of Radiology Shanghai Jiao Tong University Affiliated Sixth People's Hospital Shanghai Jiao Tong University School of Medicine 600 Yi Shan Road Shanghai 200233 China; ^2^ School of Chemistry and Chemical Engineering Frontiers Science Center for Transformative Molecules Shanghai Jiao Tong University 800 Dongchuan Road Shanghai 200240 China

**Keywords:** atherosclerosis, LOX‐1, plaque targeting, rapamycin, spherical nucleic acids

## Abstract

Atherosclerosis with unstable plaques is the dominant pathological basis of lethal cardio‐cerebrovascular diseases, which can cause acute death due to the rupture of plaques. Plaque‐targeted drug delivery to achieve promoted treatment remains the main challenge because of the systemic occurrence of atheroma. Herein, a rapamycin (RAP) spherical nucleic acid (SNA) structure, capable of specifically accumulating in plaques for synergistic atherosclerosis treatment is constructed. By designing consecutive phosphorothioate (PS) at 3’ terminus of the deoxyribonucleic acid (DNA) strand, multiple hydrophobic RAPs are covalently grafted onto the PS segment to form an amphiphilic drug‐grafted DNA (RAP‐DNA), which successively self‐assembles into micellar SNA (RAP‐SNA). Moreover, the phosphodiester‐DNA segment constitutes the outer shell of RAP‐SNA, enabling further hybridization with functional siRNA (targeting lectin‐like oxidized low‐density lipoprotein receptor‐1, LOX‐1) to obtain the drug codelivered SNA (LOX‐1/RAP‐SNA). With two active ingredients inside, LOX‐1/RAP‐SNA can not only induce robust autophagy and decrease the evil apoptosis of the pathological macrophages, but also simultaneously prohibit the LOX‐1‐mediated formation of damageable foam cells, realizing the effect of synergistic therapy. As a result, the LOX‐1/RAP‐SNA significantly reduces the progression of atheroma and stabilizes the plaques, providing a new strategy for synergistically targeted atherosclerosis treatment.

## Graphical abstract

By covalently drug grafting and supramolecularly self‐assembling, a rapamycin (RAP) spherical nucleic acid (RAP‐SNA) is constructed to hybridize with lectin‐like oxidized low‐density lipoprotein receptor‐1 (LOX‐1) siRNA to obtain the drug codelivery nanoformulation (LOX‐1/RAP‐SNA), which significantly suppresses abnormal cell apoptosis by inducing robust autophagy of macrophages and simultaneously inhibits LOX‐1‐mediated foam cell formation, resulting in synergistic antiatherosclerosis efficacy.

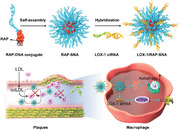

## 1 Introduction

Obstructive cardiovascular and cerebrovascular diseases caused by atherosclerosis plaque rupture is the leading cause of death worldwide. Atherosclerosis (AS) is a chronic inflammation process of the artery wall, which is characterized by lipid deposition, inflammatory cell aggregation, and extracellular‐matrix formation, resulting in the thickness of the artery wall and the formation of an unstable plaque.^[^ ^1^, ^2^, ^3^ ^]^ Currently, the clinical solutions to AS include surgical intervention and medication treatments using hypolipidemic drugs (e.g., statins), antiplatelet agents (e.g., aspirin), and peroxisome proliferator‐activated receptor‐*γ* (PPAR*γ*) agonists, etc. These clinical therapy methods always lead to high‐cost with inevitable restenosis and stent thrombosis formation (20–30% prevalence within 6 months), making it impossible for long‐term therapy.^[^ ^4^ ^]^ The mammalian target of rapamycin (mTOR) inhibitors such as rapamycin (RAP) and its analogous‐coated stents could decrease the restenosis by inhibiting the vascular inflammation and promoting abnormal macrophage clearance through autophagy‐mediated death.^[^ ^5^, ^6^ ^]^ Nonetheless, local application of stent cannot meet the clinical requests since AS is a systemic disease. In addition, drugs for medication treatments have many issues, such as off‐target effect, poor solubility, low bioavailability, short blood circulation, and nonspecific drug distribution, resulting in inefficient efficacy, long‐term administration, and severe adverse effects.^[^ ^7^, ^8^, ^9^, ^10^ ^]^ Therefore, it is highly desirable to develop new therapeutic strategies for the systemic treatment of atherosclerosis (AS).

In the past few decades, nanomedicines have obtained impressive outcomes in improving drug solubility, bioavailability, and blood circulation.^[^ ^11^ ^]^ Particularly, a variety of nanomedicines have been developed for AS treatment, including liposomes,^[^ ^12^ ^]^ polymeric nanoparticles,^[^ ^13^ ^]^ and inorganic nanomaterials.^[^ ^14^ ^]^ To achieve plaque‐targeted drug delivery and promoted therapeutic efficiency, active targeting moieties such as peptides and antibodies^[^ ^15^, ^16^ ^]^ and biomimetic formulations deriving from platelets^[^ ^17^ ^]^ and macrophage membranes^[^ ^18^, ^19^ ^]^ have to be applied in these nanomedicines. Unfortunately, these active targeting moieties lead to severe immunotoxicity^[^ ^20^ ^]^ and have unsettled drawbacks such as lack of efficient production, high heterogeneity, easy loss of cell membrane integrity during extraction, and early drug release,^[^ ^21^ ^]^ etc., which imped their expansion in clinical usage. As such, it is appealing to seek new plaque‐targeted nanoformulations with excellent biosafety for remarkable AS treatment.

**Figure 1:**
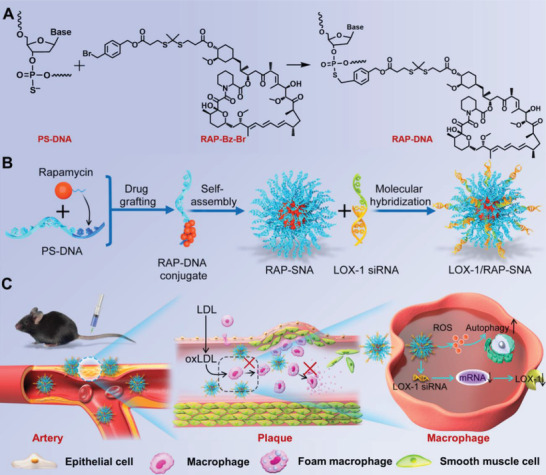
Schematic illustration for syntheses of RAP‐grafted DNA conjugate and self‐assemblies of RAP‐loaded SNAs. A) Chemical structure and synthetic route of RAP‐DNA conjugate. B) Self‐assembling of RAP‐SNA and hybridization with LOX‐1 siRNA for preparation of drug co‐packaging LOX‐1/RAP‐SNA. C) The in vivo antiatherosclerosis mechanism of LOX‐1/RAP‐SNA.

As a natural biomacromolecule with excellent biocompatibility and biodegradability, deoxyribonucleic acids (DNA) material‐based nanostructures including tetrahedrons,^[^ ^22^, ^23^, ^24^ ^]^ origamis,^[^ ^25^ ^]^ spherical nucleic acids (SNAs),^[^ ^26^ ^]^ and nanogels^[^ ^27^ ^]^ have been widely used to deliver small molecule drugs and biomacromolecules. Notably, it has been reported that oligonucleotide‐functionalized nanoparticles can promote the delivery to plaques by targeting foam macrophages through a scavenger receptor‐mediated pathway,^[^ ^28^, ^29^, ^30^ ^]^ providing a new direction in targeted AS treatment. Meanwhile, the cell internalization of SNA has been well‐studied as a scavenger receptor‐dependent endocytosis,^[^ ^31^ ^]^ thus making SNA an ideal vehicle for plaque‐targeted drug delivery and AS treatment. Herein, we construct an SNA‐based nanomedicine loading with rapamycin (RAP) to achieve plaque targeting and systemic treatment of AS. Furthermore, given the complicated pathological mechanism of AS, we propose a drug codelivery strategy by simultaneously loading small molecular RAP and gene‐drug targeting lectin‐like oxidized low‐density lipoprotein receptor‐1 (LOX‐1 siRNA) in SNA to realize the synergistic treatment of AS (**Figure** **1**). Briefly, the RAP is first covalently grafted onto DNA strand at the predesigned phosphorothioate (PS) sites to obtain an amphiphilic DNA‐drug conjugate (RAP‐DNA), which further self‐assembles into the micellar SNA structure (RAP‐SNA) with a hydrophobic drug core and oligonucleotide shell. The oligonucleotide shell maintains the base‐pairing property, which enables further integration with functional LOX‐1 siRNA designed with a sticky end at the sense strand, thereby obtaining the RAP and LOX‐1 siRNA codelivered nanomedicine (LOX‐1/RAP‐SNA). The as‐prepared LOX‐1/RAP‐SNA exhibits remarkable plaque targeting and synergistic atheroprotective efficiency. On the one hand, the RAP induces vigorous autophagy in macrophages and inhibits cell proliferation, resulting in more stabilized plaques. On the other hand, the siRNA could simultaneously downregulate the expression of LOX‐1 and reduce the engulfing of oxidized low‐density lipoprotein (oxLDL) in cells, prohibiting the formation of foam cells, thus synergistically causing regression of atheroma.

## 2 Results and Discussion

### 2.1 Syntheses, Self‐Assembly, and Characterizations of RAP‐Grafted DNA Conjugate (RAP‐DNA)

**Figure 2:**
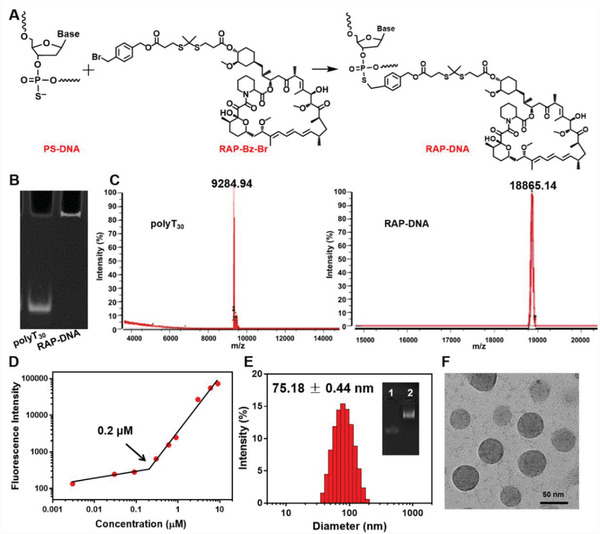
Syntheses and characterizations of RAP‐DNA conjugates and RAP‐SNA assemblies. A) Chemical synthetic route of RAP grafting on PS‐DNA strands. B) 15% denaturing polyacrylamide gel electrophoresis results of free polyT_30_ strands and RAP‐DNA conjugates. C) The MALDI‐TOF spectrum images and the observed m/z values of polyT_30_ and RAP‐DNA conjugates. D) The critical micelle concentration (CMC) of RAP‐DNA conjugate determined by a fluorescence‐based method using Nile Red as a probe. Nile red was incubated with RAP‐DNA in PBS solution at different concentrations. The obtained CMC value was 0.2 × 10^−6^ m in terms of DNA. E) The determined hydrodynamic diameter of RAP‐SNA analyzed by DLS. Inset: 1% agarose gel image of polyT_30_ strand 1) and RAP‐SNA 2). F) The morphology of RAP‐SNA nanoparticles determined by TEM. Scale bar: 50 nm.

To synthesize the amphiphilic RAP‐grafted DNA conjugates, the polyT_30_ sequence with consecutive 10 PS modifications at the 3’ terminus of the DNA strand was chosen (Supporting Information, Table S1). To graft with the PS‐DNA, the RAP was first modified with benzyl bromide group (RAP‐Bz‐Br) through 5 steps (Figure S1, Supporting Information). It is well known that the production of reactive oxygen species (ROS) generated by macrophages and smooth muscle cells (SMCs) is increased in AS lesions, resulting in an excessive oxidative environment of plaque.^[^ ^32^ ^]^ Therefore, a ROS‐responsive thioketal group^[^ ^33^ ^]^ was introduced as a linker in the syntheses of RAP‐Bz‐Br (Figure S1, Supporting Information) to realize the stimulated drug release. The structures of related compounds were characterized by hydrogen and carbon nuclear magnetic resonance spectra (^1^H‐NMR and ^13^C‐NMR, Figures S2–S5, Supporting Information), in which all peaks could be well ascribed to the targeted products. The mass spectrometry (MS) of the final RAP‐Bz‐Br exhibits the m/z values of 1347.59/1349.59 ([M+NH_4_]^+^). Afterward, the obtained RAP‐Bz‐Br was mixed with PS‐modified polyT_30_ and reacted for 1 h at 50 ℃ to synthesize the RAP‐DNA conjugate (**Figure** **2**A). Free RAP was extracted by ethyl acetate to obtain the purified RAP‐DNA, which further dried under a rotary evaporator and analyzed by multiple methods. First, 15% polyacrylamide gel (PAGE) electrophoresis was applied to characterize the mobility of RAP‐DNA conjugate. As shown in Figure 2B, the RAP‐DNA conjugate is stuck in the loading well, indicating the formation of large assemblies after PS‐DNA conjugating with hydrophobic RAP drug. Furthermore, the molecule weight of RAP‐DNA conjugate was investigated by matrix‐assisted laser desorption/ionization‐time of flight mass spectrometry (MALDI‐TOF). The observed m/z values of polyT_30_ and RAP‐DNA are 9284.94 and 18 865.14 (Figure 2C), respectively, indicating ≈8 RAPs are conjugated on a DNA strand, giving a drug loading content of 50.78%. Next, we further investigated the amphipathicity and assembly property of RAP‐DNA by determining the critical micelle concentration (CMC) in phosphate buffer saline (PBS) using Nile Red as the fluorescent probe (Figure 2D). The RAP‐DNA conjugate has a CMC value of 0.2 × 10^−6^ m (in terms of DNA), revealing the amphipathicity of RAP‐DNA, which can retain in the aggregated state at a low concentration. Later on, we prepared the RAP‐loading SNA (RAP‐SNA) using the dialysis method and characterized the assembly by 1% agarose gel electrophoresis (Figure 2E). The RAP‐SNA exhibits a sharp band with a lower mobility than the polyT_30_ strand in agarose gel, indicating the successful assembly of RAP‐SNA with excellent uniformity. Additionally, the hydrodynamic diameter of the RAP‐SNA was measured by dynamic laser scattering (DLS). As shown in Figure 2E, the observed diameter of RAP‐SNA is 75.18 ± 0.44 nm with a polydispersity index of 0.1. The assembled RAP‐SNA shows a spherical morphology as observed by transmission electron microscope (TEM, Figure 2F) and atomic force microscope (AFM, Figure S6, Supporting Information). These above results indicate the successful assembly of RAP‐loaded SNAs. Furthermore, the physiological stability of RAP‐SNA was investigated by incubating with 10% fetal bovine serum (FBS)‐containing buffer and 5 U mL^−1^ DNase I for varied time intervals and then characterized the products by 0.5% agarose gel electrophoresis under native condition. As shown in Figure S7A (Supporting Information), no obvious change can be observed of the SNAs after incubation with 10% FBS or 5 U mL^−1^ DNase I for 12 h, indicating the excellent stability of SNAs during blood circulation. Moreover, to investigate the ROS‐responsive drug release property, the compound RAP‐Bz‐Br and RAP‐SNA nanomedicine were cocultured with hydrogen peroxide (H_2_O_2_) for further characterizations. After incubating the RAP‐Bz‐Br with H_2_O_2_, a new band appeared with higher polarity in the thin layer chromatography (TLC) plate, indicating the cleavage of the ROS‐responsive thioketal linker (Figure S8A, Supporting Information). Moreover, the RAP‐SNA nanomedicine exhibits a ROS‐stimulated drug release that 95% RAPs are released from the SNA after incubating in 1 × 10^−3^ m H_2_O_2_ for 24 h, while only 40% RAPs are released under PBS condition (Figure S8B, Supporting Information).

### 2.2 In Vitro Cellular Uptake of RAP‐SNA

**Figure 3:**
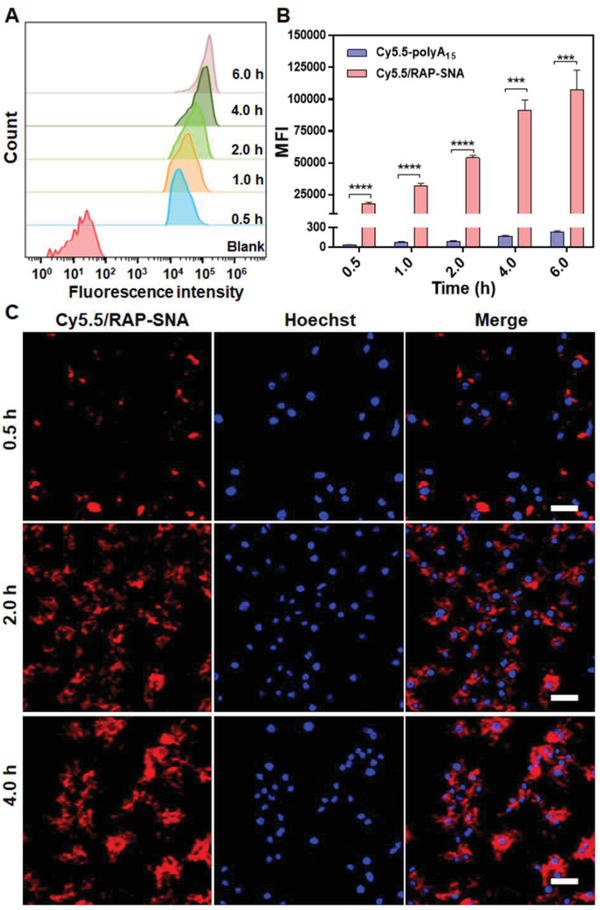
In vitro cellular uptake of Cy5.5‐labeled RAP‐SNA (Cy5.5/RAP‐SNA). A) Flow cytometry analysis after incubating Cy5.5/RAP‐SNA with RAW264.7 cells for different time intervals. B) The summarized mean fluorescence intensities (MFI) of the RAW264.7 cells after incubating with Cy5.5‐polyA_15_ and Cy5.5/RAP‐SNA for different time intervals. C) CLSM images of RAW264.7 cells after incubating with Cy5.5/RAP‐SNA for 0.5, 2.0, and 4.0 h, respectively. Scale bar: 25 µm. Statistical significance: ***, *P* < 0.001; ****, *P* < 0.0001.

Following the self‐assembly of RAP‐SNA, we further investigated the cell uptake behaviors of RAP‐SNA in macrophages (RAW264.7) by flow cytometry and confocal laser scanning microscopy (CLSM) imaging. The cyanine5.5‐labeled RAP‐SNA (Cy5.5/RAP‐SNA) was prepared by hybridizing Cy5.5‐labeled polyA_15_ strands (Cy5.5‐polyA_15_) onto RAP‐SNA through Watson‐Crick base pairings, which exhibits a sharp band with lower mobility than original RAP‐SNA in 0.5% agarose gel (Figure S9, Supporting Information). Thereafter, the Cy5.5/RAP‐SNA was incubated with RAW264.7 cells for different time intervals at a concentration of 0.5 × 10^−6^ m (Cy5.5) and then analyzed by flow cytometry. As shown in **Figure** **3**A,B, the Cy5.5/RAP‐SNA remarkably enhances the internalization into macrophages compared with the free Cy5.5‐polyA_15_ strand, which could be ascribed to the particular 3D structure of SNA.^[^ ^34^ ^]^ In addition, the Cy5.5/RAP‐SNA exhibits a time‐dependent cell uptake behavior, as evidenced by the enhanced fluorescence intensity when incubating for a longer time. Furthermore, the intracellular distribution of Cy5.5/RAP‐SNA was visualized by CLSM imaging after incubated with the RAW264.7 cells for 0.5, 2.0, and 4.0 h, respectively. As shown in Figure 3C, the Cy5.5/RAP‐SNA well‐distributes inside RAW264.7 cells, as evidenced by the obvious fluorescence signal in cells. Moreover, the cells have an enhanced Cy5.5/RAP‐SNA signal when prolonged the incubation time, indicating the time‐dependent cellular internalization of SNAs, which is consistent with the flow cytometry results.

### 2.3 In Vitro Autophagy Induction and LOX‐1 Downregulation in Macrophages

**Figure 4:**
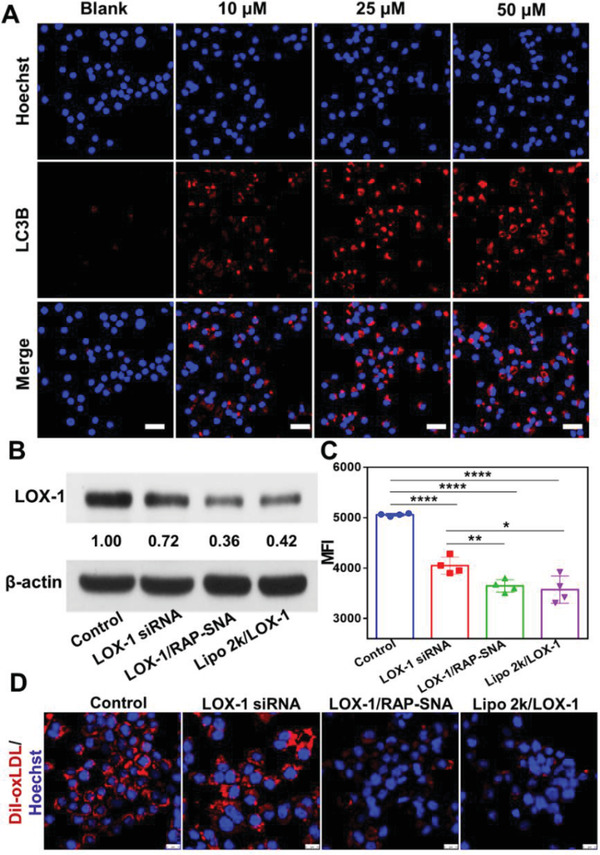
In vitro therapeutic evaluation of RAP‐SNA and LOX‐1/RAP‐SNA. A) The CLSM images of RAW264.7 cells after treatment with RAP‐SNA at different RAP concentrations (10, 25, and 50 × 10^−6^ m) for 24 h and stained with anti‐LC3B (autophagy marker, red signal) antibody. Scale bar: 25 µm. B) Western blot analysis of LOX‐1 expression in RAW264.7 cells after treatment with different LOX‐1 siRNA‐loading formulations. C) Flow cytometry analysis of the engulfing of DiI‐oxLDL in LOX‐1 siRNA, LOX‐1/RAP‐SNA, and Lipo 2k/LOX‐1 siRNA‐treated RAW264.7 cells. D) CLSM images of the different LOX‐1 siRNA formulations‐treated RAW264.7 cells after incubation with DiI‐oxLDL for 5 h. Scale bar: 10 µm. Statistical significance: **, *P* < 0.01; ***, *P* < 0.001; ****, *P* < 0.0001.

It is well‐known that macrophages are the central character in atherosclerosis progression by proliferation, secreting of proinflammatory mediators, and synthesis of metalloproteinase in plaque lesion.^[^ ^35^, ^36^ ^]^ The macrophage autophagy becomes deficient in advanced AS, leading to the sustained progression of the disease.^[^ ^37^ ^]^ It was reported that inducing autophagy in macrophages could promote the stability of plaques and attenuate the severity of AS.^[^ ^38^, ^39^ ^]^ Since RAP is a well‐studied autophagy inducer, accordingly, we examined the autophagy induction capacity of RAP‐SNA in RAW264.7 cells by detecting the LC3B protein (a marker of autophagy) using the immunofluorescence technique. After the RAW264.7 cells were treated with RAP‐SNA for 24 h at different RAP concentrations (10, 25, and 50 × 10^−6^ m), the anti‐LC3B antibody and fluorescent secondary antibody were sequentially added to visualize the LC3B‐specific fluorescence signals by CLSM imaging. As shown in **Figure** **4**A, the RAP‐SNA induces robust autophagy in macrophages, as evidenced by the strong fluorescence signals observed in the RAP‐SNA‐treated RAW264.7 cells, revealing the therapeutic potential of RAP‐SNA in AS management. Besides as an autophagy inducer, the RAP can also inhibit cell proliferation.^[^ ^40^ ^]^ Therefore, the antiproliferation effect of RAP‐SNA was evaluated in RAW264.7 cells by evaluating the cell viability and cell counts after incubation with RAP‐SNA for 24 h (Figures S10 and S11, Supporting Information). The RAP‐SNA significantly inhibits the macrophage proliferation after incubating for 24 h as evidenced by the decreased cell viability and cell counts number.

In the progress of AS, macrophages in plaques will turn into foam cells by aberrantly engulfing oxidized low‐density lipoproteins (oxLDL) through overexpressed lectin‐like ox‐LDL receptor‐1 (LOX‐1),^[^ ^36^, ^41^ ^]^ which is one of the leading causes of the instability and fragility of plaque. Foam cells are prone to apoptosis and release tissue factors and lipids, which cannot be sufficiently cleared due to the defective efferocytosis effect of the pathological marcophages,^[^ ^42^ ^]^ resulting in the formation of a necrotic core and instability of plaques. Though the RAP could reduce the abnormal macrophages,^[^ ^5^ ^]^ it cannot effectively prevent the formation of vulnerable foam cells. Therefore, combining RAP with drugs that inhibit the formation of foam cells such as LOX‐1 blockers may have a synergistic treatment efficiency for AS. Herein, we chose the siRNA drug as a LOX‐1 blocker to inhibit the formation of foam cells by decreasing the engulfing of oxLDL. We prepared the RAP and LOX‐1 siRNA codelivered formulations (LOX‐1/RAP‐SNA) by designing a polyA overhang on the sense strand of siRNA (Table S1, Supporting Information) to associate with the RAP‐SNA. The LOX‐1 siRNA loading capacity of RAP‐SNA was investigated using 0.5% agarose gel electrophoresis by changing the ratio of RAP‐SNA/siRNA (Figure S12, Supporting Information). The band of the LOX‐1/RAP‐SNA is obtained in agarose gel when the RAP‐SNA/siRNA ratios change from 1:0.05 to 1:0.2, while the free siRNA band appears when the ratio reaches 1:0.25, indicating the RAP‐SNA has a maximum siRNA loading capacity of 20%. In addition, the LOX‐1/RAP‐SNA exhibits excellent physiological stability for at least 12 h as evidenced by the similar bands of 10% FBS‐ and DNase I‐treated SNAs with nontreated SNAs (Figure S7B, Supporting Information). Next, the LOX‐1 downregulation by LOX‐1/RAP‐SNA in macrophages was evaluated using western blot assay. The RAW264.7 cells were transfected with free LOX‐1 siRNA, LOX‐1/RAP‐SNA, and lipofectamine 2000‐transfected LOX‐1 siRNA (Lipo 2k/LOX‐1) for 12 h at a siRNA concentration of 50 × 10^−9^ m, followed by refreshing the medium with Dulbecco's modified eagle medium (DMEM) and incubation for another 36 h. Total proteins were collected for western blot analysis. As shown in Figure 4B, compared with that in control and free LOX‐1 siRNA‐treated cells, the expression of LOX‐1 protein in macrophages significantly decreases in LOX‐1/RAP‐SNA‐treated cells, which could be ascribed to the enhanced cell internalization of the nanoparticles. Sequentially, we further explored the oxLDL engulfing in LOX‐1‐downregulated macrophages by incubating the DiI‐labeled oxLDL (DiI‐oxLDL) with LOX‐1/RAP‐SNA‐treated cells for flow cytometry (FCM) analysis. As shown in Figure 4C, the untreated macrophages have an excellent DiI‐oxLDL engulfing capacity as evidenced by the high mean fluorescent intensity (MFI) observed by flow cytometry (control group). Note that, after being treated with LOX‐1 siRNA and LOX‐1/RAP‐SNA, the uptake of DiI‐oxLDL by macrophages is significantly reduced due to the downregulation of LOX‐1 receptors, implying the potential therapeutic efficiency of LOX‐1 siRNA. In addition, to further verify this conclusion, the THP‐1 cell‐derived macrophages were used to perform experiments. Likewise, the uptake of DiI‐oxLDL by THP‐1 derived cells remarkably decreases after treatment with LOX‐1/RAP‐SNA and Lipo 2k/LOX‐1 (Figure S13, Supporting Information), indicating the reduction of LOX‐1 dependent oxLDL uptake. Furthermore, CLSM was used to visually investigate the uptake of ox‐LDL in RAW264.7 cells (Figure 4D). In consistent with the FCM results, the fluorescence intensity of the DiI signal significantly reduces after cells were treated with LOX‐1/RAP‐SNA and Lipo 2k/LOX‐1, indicating the downregulation of LOX‐1 decreases the engulfing of oxLDL by macrophages, which would prohibit the foam cell formation.

### 2.4 Plaque‐Targeted Accumulation of RAP‐SNA and In Vivo Magnetic Resonance (MR) Imaging

**Figure 5:**
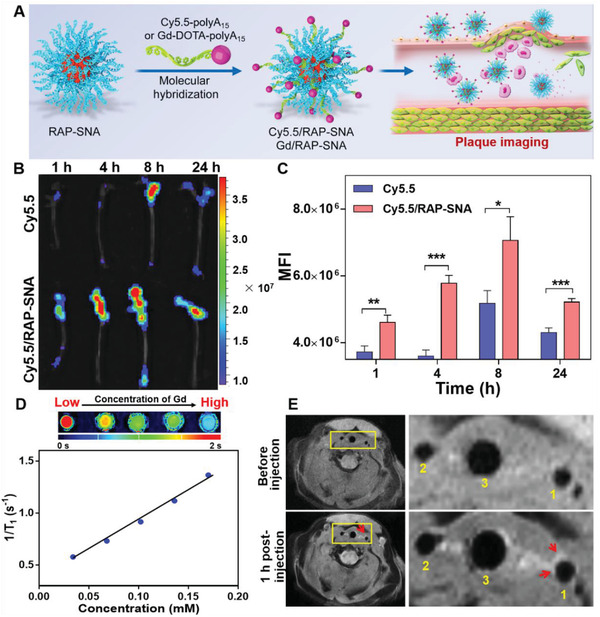
In vivo plaque accumulation and imaging ability of Cy5.5/RAP‐SNA and Gd/RAP‐SNA. A) Preparation of the imaging formulations by molecular hybridization between RAP‐SNA and Cy5.5‐ and Gd‐DOTA‐modified polyA_15_ strands in PBS buffer. B) Ex vivo fluorescent imaging of the aortas obtained from ApoE^–/–^ mice at 1, 4, 8, and 24 h postinjection of free Cy5.5 solution and Cy5.5/RAP‐SNA. C) The mean fluorescence intensity (MFI) of the imaged aortas. D) T_1_‐mapping of Gd/RAP‐SNA at different concentrations and the regression curve between the 1/T_1_ and concentration of Gd. The calculated relaxation rate of Gd/RAP‐SNA is 5.8 × 10^−3^ m s^−1^. E) In vivo MRI images of the carotid recorded before and 1 h postinjection of Gd/RAP‐SNA. 1) left common carotid artery; 2) right common carotid artery; 3) trachea. Statistical significance: *, *P* < 0.05; **, *P* < 0.01; ***, *P* < 0.001.

According to the previous study, the SNA structure could specifically accumulate in plaque lesion,^[^ ^28^ ^]^ which would exert excellent therapeutic efficacy. To investigate whether the prepared RAP‐SNA could improve the accumulation in AS lesion, Cy5.5/RAP‐SNA was prepared (**Figure** **5**A) and intravenously injected via tail vein into the apolipoprotein E gene‐knockdown (ApoE^−/−^) mice using free Cy5.5 solution as control. After different time intervals (1.0, 4.0, 8.0, and 24 h), the atherosclerotic mice were anaesthetized and went through heart perfusion to separate aorta for ex vivo fluorescence imaging. As shown in Figure 5B, in comparison with free Cy5.5 solution, the Cy5.5/RAP‐SNA can rapidly distribute in plaques within 1 h and the fluorescent signals in aortas maintain a remarkable level even at 24 h postinjection, demonstrating the Cy5.5/RAP‐SNA can sustainably accumulate at plaque area. In addition, according to the fluorescent quantitative results (Figure 5C), the aortas in Cy5.5/RAP‐SNA‐treated mice exhibit stronger fluorescence intensities at all the time points than that of free Cy5.5, indicating the SNA structure can significantly improve drug accumulation in plaques. The above results confirmed the plaque targeting ability of our prepared RAP‐SNA formulations. To understand the bio‐distribution of free drugs and SNA formulations, main tissues were simultaneously collected for fluorescence imaging. As shown in Figure S14 (Supporting Information), the free Cy5.5 mainly distributes in liver and kidneys and no obvious signals are observed in liver after 24 h, indicating that the free drug could be eliminated by liver. However, kidneys exhibit strong drug signals even after 24 h, revealing free drugs can be enriched in kidneys, which might result in kidney damages. In comparison, the Cy5.5/RAP‐SNA mainly distributes in liver but not kidneys, demonstrating the SNA would reduce the damage risk in kidneys. After 24 h postinjecting Cy5.5/RAP‐SNA, obvious signals can be observed in liver, indicating the long‐circulating property of SNAs. To further investigate the fluorescence distribution inside the plaques, the aortas collected from mice ApoE^−/−^ mice after intravenously injecting Cy5.5/RAP‐SNA were made into frozen cross‐sections and stained with nucleus dye 4', 6‐diamidino‐2‐phenylindole (DAPI) for CLSM imaging. As shown in Figure S15 (Supporting Information), the Cy5.5/RAP‐SNA mainly distributes inside plaques as evidenced by the obvious fluorescent signals in intima area and the medium of the artery. The results demonstrate the exceptional plaque accumulation ability and long circulation of RAP‐SNA, which ensures the therapeutic efficacy for AS treatment.

Additionally, the RAP‐SNA can be further applied for the in vivo magnetic resonance imaging (MRI) to achieve the diagnosis functionality by simply replacing the Cy5.5‐polyA_15_ with the contrast agent‐modified polyA_15_ strand. As a proof of concept, therefore, we first synthesized a DOTA (1,4,7,10‐tetraazacyclododecane‐1,4,7,10‐tetraacetic acid)‐polyA_15_ conjugate using amino‐modified polyA_15_ (polyA_15_‐NH_2_) and 1,4,7,10‐tetraazacyclododecane‐1,4,7,10‐tetraacetic acid mono‐N‐hydroxysuccinimide ester (DOTA‐NHS), which can be coordinated with gadolinium ion (Gd^3+^) to obtain the Gd‐DOTA‐polyA_15_ (Figure S16, Supporting Information). The successful syntheses of DOTA‐polyA_15_ and Gd‐DOTA‐polyA_15_ were characterized by 20% denaturing PAGE electrophoresis (Figure S17A, Supporting Information). The DOTA‐polyA_15_ exhibits a sharp band with lower mobility than unconjugated polyA_15_, indicating the complete conjugation between polyA_15_‐NH_2_ and DOTA‐NHS. After mixing DOTA‐polyA_15_ with Gd^3+^ ion, a new band appears above the DOTA‐polyA_15_, revealing the successful preparation of Gd‐DOTA‐polyA_15_. Next, similar to the assembly of Cy5.5/RAP‐SNA, the Gd‐loaded SNA (Gd/RAP‐SNA) was prepared by hybridizing the Gd‐DOTA‐polyA_15_ onto RAP‐SNA in PBS solution via base‐pairing at room temperature. As characterized by 0.5% agarose gel electrophoresis (Figure S17B, Supporting Information), the Gd/RAP‐SNA exhibits lower mobility than RAP‐SNA, indicating the Gd‐DOTA‐polyA_15_ is successfully hybridized onto RAP‐SNA. Thereafter, we investigated the T_1_ relaxation time of the prepared Gd/RAP‐SNA in water solutions at different gadolinium concentrations by T_1_ mapping on a 3T MAGNETOM Prisma MRI Scanner (Siemens Healthineers, Figure 5D). Furthermore, by regression between 1/T_1_ and the concentration of Gd, the relaxation rate is obtained. The calculated relaxation rate is 5.8 × 10^−3^ m s^−1^ (Figure 5D), indicating the MRI ability of Gd/RAP‐SNA. Later on, the Gd/RAP‐SNA was used for in vivo MRI on atherosclerotic ApoE^−/−^ mouse model. The in vivo MR imaging was performed on an 11.7 T BioSpec MRI system before and 1 h postinjection of the Gd/RAP‐SNA to detect the carotid area. As shown in Figure 5E and Figure S18 (Supporting Information), an obvious heterogeneous enhancement in the left common carotid artery wall is observed at 1 h postinjection of Gd/RAP‐SNA compared with that before‐injection, indicating the formation of plaques in this area. The results demonstrate the potential of SNA in the MRI diagnosis of atherosclerosis, providing a new horizon in the application of DNA nanostructure.

### 2.5 In Vivo Therapeutic Efficacy in Atherosclerotic Mice

**Figure 6:**
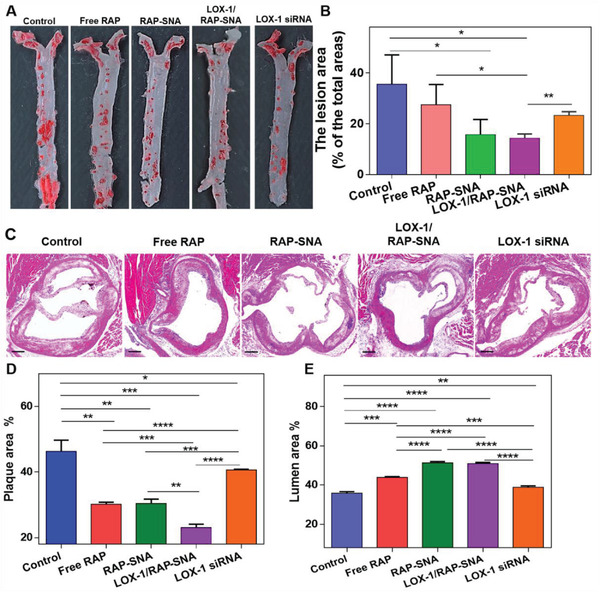
In vivo antiatherosclerosis efficiency. A) ORO‐stained images of aortas dissected from ApoE^−/−^ mice after treatment with free RAP, RAP‐SNA, LOX‐1/RAP‐SNA, and LOX‐1 siRNA for 4 weeks. B) Quantitative analysis of the plaque lesion in the ORO‐stained images. C) H&E‐stained images of the aortas roots cross‐section obtained from ApoE^−/−^ mice after treatment with different formulations. D,E) Statistical analysis of the plaque D) and lumen E) areas in H&E‐stained images. Statistical significance: *, *P* < 0.05; **, *P* < 0.01; ***, *P* < 0.001; ****, *P* < 0.0001. Scale bar: 200 µm.

To investigate the antiatherosclerosis efficacy of LOX‐1/RAP‐SNA, the ApoE^−/−^ mice were fed with a high‐fat diet for 8 weeks to establish the atherosclerotic model. Thereafter, the atherosclerotic mice were intravenously injected with different drug formulations, including free RAP solution, RAP‐SNA, LOX‐1/RAP‐SNA, and LOX‐1 siRNA twice a week for 7 times at a dosage of 4 mg RAP kg^−1^ mice and 0.5 mg siRNA kg^−1^ mice. Mice without any treatment were utilized as the control group. As the therapy finished, aortas were dissected for oil red O (ORO) staining to visualize the size of plaques after the mice were anaesthetized. As shown in **Figure** **6**A,B, atherosclerotic mice in the control group exhibit severe AS progression with the largest plaque area, about 33.6% of the total aorta. After treatment with free RAP and free LOX‐1 siRNA, the proportions of plaque area are decreased to 27.5% and 23.3%, respectively, indicating the RAP and LOX‐1 siRNA could attenuate the progression of AS. Compared with free RAP solution, the RAP‐SNA and LOX‐1/RAP‐SNA formulations remarkably reduce the plaque proportions to 15.7% and 14.3% after treatment, demonstrating that the SNA structure could improve the anti‐atherosclerosis efficiency of RAP, which can ascribe to the enhanced drug accumulation in plaques. Furthermore, the aortas roots were made into cross‐sections and stained with hematoxylin and eosin (H&E) to analyze the aortic pathology morphology. As shown in Figure 6C, the LOX‐1/RAP‐SNA treated mice exhibit the least atherosclerotic pathology compared with that in other groups, demonstrating the remarkable synergistic therapeutic effect of the drug coloading formulation. In addition, the proportions of plaque and lumen to the aorta in the H&E stained sections were statistically calculated to analyze the antiatherosclerosis efficiency of different formulations. As shown in Figure 6D,E, compared with control group (with 46.3% plaque area and 35.8% lumen area), the free RAP solution and RAP‐SNA could remarkably inhibit the plaque progression, as evidenced by the reduced plaque percentages (30.2% and 30.4%) and enhanced lumen percentages (43.9% and 51.4%). What's more, the LOX‐1/RAP‐SNA‐treated mice exhibit the most regression of plaques, as evidenced by the minimum plaque (23.0%) and enhanced lumen area (51.0%), implying the excellent therapeutic efficiency of the LOX‐1/RAP‐SNA. To confirm our conclusion, we further analyzed the pathological sections of innominate arteries of all the groups. As shown in Figure S19 (Supporting Information), in consistent with the aorta root section, the LOX‐1/RAP‐SNA‐treated mice exhibit the most regression of plaques in innominate artery, demonstrating the excellent anti‐atherosclerosis efficiency.

**Figure 7:**
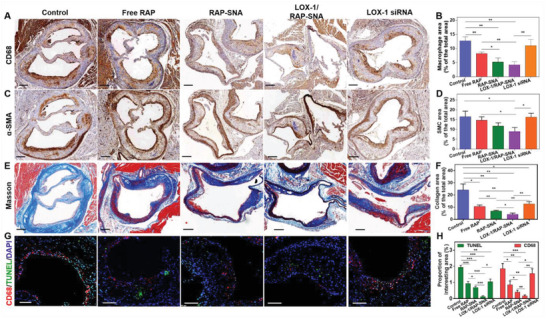
Immunohistochemistry analysis of the aortic root sections obtained from ApoE^−/−^ mice after different treatments. A) Anti‐CD68 antibody‐stained aortic root section images to evaluate the macrophages in plaques. B) Quantitative analysis of the macrophages in plaques. C) *α*‐SMA‐stained aortic root section images to analyze the SMCs in plaque. D) Quantitative analysis of the SMC in plaque. E) Masson staining images of the aortic root section to analyze the collagen amount based on the total vessel area. Scale bar: 200 µm. F) Quantitative analysis of the collagen in plaque. G) TUNEL and anti‐CD68 antibody‐stained images to evaluate the cell apoptosis in plaque. Scale bar: 100 µm. H) Quantitative analysis of apoptotic cells in plaque. Statistical significance: *, *P* < 0.05; **, *P* < 0.01; ***, *P* < 0.001; ****, *P* < 0.0001.

Furthermore, the constituents of plaques were analyzed by immunohistochemically staining the cross‐section of aortas obtained from mice with different treatments. It is well known that the macrophages accumulating and proliferating in plaque are the key events of the AS progression, which can further engulf oxidized low‐density lipoprotein (oxLDL) to form the foam cells, leading to an unstable plaque.^[^ ^43^ ^]^ Therefore, the macrophage content reflects the progression degree of AS disease. Accordingly, we stained the cross‐section of the aortas with anti‐CD68 (a typical marker of macrophages) antibodies to investigate the macrophage content in plaques. As shown in **Figure** **7**A,B, compared with control group (13.29% macrophage) after treatment with free RAP, RAP‐SNA, and free LOX‐1 siRNA, the macrophage content significantly reduces to 7.83%, 5.83%, and 11.73%, respectively. The LOX‐1/RAP‐SNA treated mice have the fewest macrophage (4.15%), implying the remarkable disease management ability of the synergistic formulation. Despite macrophages, the proliferation of smooth muscle cells (SMCs) is another event in the genesis of lesions.^[^ ^44^ ^]^ Therefore, we investigated the SMC content by staining the aortas cross‐section with anti‐*α*‐smooth muscle actin (*α*‐SMA, a marker of SMC) antibody. As shown in Figure 7C,D, compared with control groups (16.54% SMC), the SMC content reduces to 14.68% and 11.69% after treatment with free RAP and RAP‐SNA, while no significant decrease is observed after treatment with free LOX‐1 siRNA (16.24%). The LOX‐1/RAP‐SNA treated mice show the fewest SMC content with a proportion of 8.95%, indicating its excellent attenuation of atheroma. In addition, the collagen content (Figure 7E,F) significantly decreases to 4.05% in the LOX‐1/RAP‐SNA‐treated group, further indicating the remarkable antiatherosclerosis efficacy of the synergistic formulation. It has been reported that the apoptosis of damageable foam cells coupled with defective phagocytic clearance of the apoptotic cells is crucial for the development of unstable plaque.^[^ ^42^, ^45^, ^46^ ^]^ Therefore, we analyzed the apoptotic cells in plaques by the TdT‐mediated dUTP nick end labeling (TUNEL) approach to evaluate the plaque stability. As shown in Figure 7G,H, in mice without any treatment, vast apoptotic cells are observed in the intima of the artery, which implies the vulnerability of the fabric cap, demonstrating an unstable plaque property. After treatments with free RAP‐SNA, RAP‐SNA, and LOX‐1 siRNA, the apoptosis rate significantly decreases, revealing the improved stability of plaques. Moreover, the LOX‐1/RAP‐SNA treatment group exhibits the most stabilized plaques, as evidenced by the fewest apoptosis. All the results demonstrate that the drug codelivered LOX‐1/RAP‐SNA can significantly stabilize the plaques and promote the regression of AS, which might be ascribed to the enhanced autophagy and LOX‐1 downregulation‐caused decrease of oxLDL engulfing. To further validate the assumption, the autophagy and LOX‐1 expression in plaques were evaluated by immunofluorescence staining after the treatment with different formulations. As shown in Figure S20 (Supporting Information), the LOX‐1/RAP‐SNA induces remarkable autophagy and significantly downregulates the expression of LOX‐1 in plaques as evidenced by the enhanced LC3B signals and decreased LOX‐1 signals, providing a validation for the effective antiatherosclerosis efficiency of LOX‐1/RAP‐SNA.

### 2.6 In Vivo Biosafety Assessment

**Figure 8:**
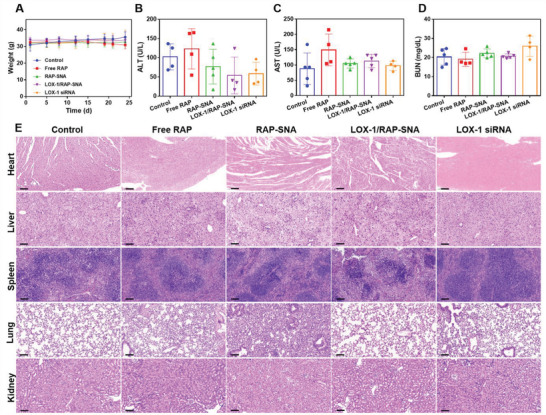
In vivo bio‐safety evaluation of different drug formulations. A) The recorded weight of ApoE^−/−^ mice during the treatment process. B–D) The biochemical analysis of blood obtained from mice after different treatments to evaluate the liver and kidney function. ALT, alanine aminotransferase; AST, aspartate aminotransferase; BUN, blood urea nitrogen. E) H&E‐stained images of heart, liver, spleen, lung, and kidney tissues obtained from the mice after being treated with different drug formulations for 4 weeks. Scale bar: 100 µm.

To evaluate the biosafety of different formulations, the mice weights were recorded during the treatments. As shown in **Figure** **8**A, there is no significant weight change in RAP‐SNA and LOX‐1/RAP‐SNA treatment groups, indicating the biosafety of RAP‐SNA and LOX‐1/RAP‐SNA. In addition, the blood serums were harvested after treatment to detect the biochemical indexes to evaluate liver and kidney functions. As shown in Figure 8B–D, compared with the control group, the drug‐treated mice show a similar level of alanine aminotransferase (ALT), aspartate aminotransferase (AST), and blood urea nitrogen (BUN), demonstrating the functions of liver and kidney were not destroyed by these treatments. Accordingly, the H&E staining images of the heart, liver, spleen, lung, and kidney indicate no distinguishable change compared with control mice, which further confirms the biosafety and biocompatibility of the RAP‐SNA and LOX‐1/RAP‐SNA. All the results imply the potentiality of our prepared LOX‐1/RAP‐SNA as an excellent candidate for the long‐term treatment of atherosclerosis.

## 3 Conclusions

In summary, a DNA material‐based nanostructure with excellent plaque targeting property and promoted efficacy was fabricated to achieve synergistic AS treatment. Through a well‐established phosphorothioate modification approach, multiple hydrophobic RAPs were facilely anchored onto the designed PS site of DNA strands, resulting in an amphiphilic multi‐RAP grafted DNA conjugate, which could self‐assemble into micellar RAP‐SNA structure. The RAP‐SNA accumulated in plaques could induce robust autophagy in macrophages and inhibit cell proliferation through autophagy‐mediated death, thereby suppressing the abnormal cell apoptosis in plaques. In addition, by hybridizing with LOX‐1 siRNA, the codelivery system LOX‐1/RAP‐SNA was obtained, which significantly downregulates LOX‐1 expression on membranes, thus reducing the engulfing of oxLDL in macrophages and prohibiting the formation of foam cells. Consequentially, the LOX‐1/RAP‐SNA effectively prohibits the progression of atheroma in ApoE^−/−^ mice, resulting in more stabilized plaques with smaller plaque core, fewer macrophages, and lower apoptosis in the plaque area. To summarize, this study presents a facile‐manufactured DNA‐based nanoformulation for simultaneous delivery of RAP and LOX‐1 blockers with good biosafety, specific plaque targeting, and promoted anti‐atherosclerosis efficiency, providing a promising delivery platform for plaque‐targeted drug delivery and synergistic AS therapy. What's more, the as‐prepared RAP‐SNA can intrinsically accumulate in plaques and realizes in vivo observation of plaques at an early stage by MRI after loading with a contrast agent, supplying a new direction in the bio‐application of DNA nanomedicines.

## 4 Experimental Section

### Materials

DNA and RNA strands were obtained from Shanghai DNA Bioscience Co. Ltd., China. 3‐mercaptopropionic acid (MPA), hydrogen peroxide (H_2_O_2_), tetrahydrofuran (THF), dimethyl sulfoxide (DMSO), sulfuric acid (H_2_SO_4_), acetone, dicyclohexylcarbodiimide (DCC), trifluoroacetic acid, dichloromethane (DCM), dimethylaminopyridine (DMAP), ethyl acetate (EA), and petroleum ether were purchased from Sinopharm Chemical Reagent Co. Ltd. (China). Rapamycin, (4‐bromomethyl)phenyl)methanol, imidazole, trimethylsilyl chloride (TMS‐Cl), anhydrous sodium sulfate (Na_2_SO_4_), sodium bicarbonate (NaHCO_3_), and *N*,*N*'‐Diisopropylcarbodiimide (DIC) were purchased from Shanghai Bide Shanghai Bide pharmaceutical technology Co. Ltd., China. DOTA‐NHS was purchased from Shanghai Apeptide Co., Ltd., China. PBS, Opti‐MEM medium, DMEM, and fetal bovine serum (FBS) were purchased from Thermo Fisher Scientific, USA. Methyl Thiazolyl Tetrazolium (MTT), and Hoechst 33 342 were purchased from Beyotime Biotechnology, China.

### Synthesis of Benzyl Bromide‐Modified Thioketal (TK‐Bz‐Br, Compound 1)

First, a ROS cleavable thioketal (TK) was synthesized according to the previously reported method using 3‐mercaptopropionic acid and acetone.^[^ ^47^ ^]^ Thereafter, (4‐bromomethyl)phenyl)methanol (1 equivalent (eq.)) and the synthesized thioketal (3 eq.) were mixed in dry DCM followed by the addition of DMAP (0.5 eq). After stirring for 10 min, DCC dissolved in dry DCM were added dropwise under ice‐bath conditions. The reaction was monitored by thin‐layer chromatography (TLC) using EA/petroleum ether as the mobile phase. The final product was purified by silica gel column chromatography to obtain TK‐Bz‐Br (compound 1). The structure of TK‐Bz‐Br was characterized by ^1^H‐NMR and ^13^C‐NMR. Yield: 65%.

### Synthesis of Benzyl Bromide‐Modified Rapamycin (RAP‐Bz‐Br)

1 g rapamycin (RAP, 1 eq.) was dissolved in 20 mL EA. After cooling the solution to 0 ℃, imidazole (10 eq.) was added and stirred to dissolve. Thereafter, the protecting reagent TMS‐Cl (8 eq.) dissolved in 5 mL EA was added dropwise and reacted for approximate 2 h to obtain the RAP‐31,42‐*bis*‐O‐TMS. After the reaction was complete, 6 mL 0.5 m H_2_SO_4_ was dropwise added and stirred under an ice bath overnight. 60 mL EA was added into the mixture and the organic layer was successively washed with saturated NaHCO_3_ solution (twice) and deionized water (3 times) to adjust the pH of the aqueous layer to 6–7. The EA layer was dried over Na_2_SO_4_ and concentrated to obtain the RAP‐31‐O‐TMS, compound 2 (Yield: 90%). The observed m/z value of compound 2 by mass spectrum (MS) was 1008.61, [M + Na]^+^. Thereafter, the TK‐Bz‐Br (1 eq.) and RAP‐31‐O‐TMS (0.8 eq.) were mixed and dissolved in dry DCM, followed by the addition of DMAP (0.5 eq.). The DIC (2 eq.) dissolved in DCM was added and stirred overnight. The final product was purified by silica gel column chromatography to obtain RAP‐31‐O‐TMS‐42‐Bz‐Br, compound 3 (Yield: 35%). The observed m/z values of compound 3 was 1419.64/1421.64, [M + NH_4_]^+^. Compound 3 (0.4 g) was dissolved in 10 mL acetonitrile and 20 mL EA, followed by the addition of 3 mL 1 m H_2_SO_4_ and stirred for 2 h under ice bath condition. 50 mL EA was added and the organic layer was washed with water twice. The final EA layer was dried over Na_2_SO_4_ and concentrated to obtain the RAP‐Bz‐Br, compound 4. The observed m/z value of compound 4 was 1347.59/1349.59, [M + NH_4_]^+^. All the intermediates and products were characterized by ^1^H‐NMR and ^13^C‐NMR.

### Synthesis of Rapamycin‐DNA Conjugate (RAP‐DNA)

5 OD PS‐DNA was dried under rotary evaporation. Then 2.2 mg RAP‐Bz‐Br dissolved in 0.1 mL DMSO was added and vibrated under 50 ℃ for 1 h. Then, deionized water was added into the mixture and washed by EA to remove free RAP‐Bz‐Br. The final RAP‐DNA conjugate was obtained after dried over. 15% denaturing PAGE electrophoresis was used to characterize the successful synthesis of RAP‐DNA. The molecule weight of RAP‐DNA was determined by matrix‐assisted laser desorption ionization time‐of‐flight mass spectrometry (MALDI‐TOF, Shimadzu MALDI 7090, Japan). To determine the critical micelle concentration (CMC) of RAP‐DNA, 1 µL Nile Red solution (100 × 10^−6^ m) dissolved in THF was added into 100 µL RAP‐DNA solution at a concentration range from 0.003 to 9 × 10^−6^ m in terms of DNA. After incubated for 2 h, the fluorescence intensity of Nile Red was determined on a microplate reader (Synergy H4, Biotek, USA) with the excitation/emission wavelengths of 543/620 nm. The CMC value was obtained at the concentration point that induce the rush of fluorescence intensity.

### Self‐Assembly of RAP‐Loaded Spherical Nucleic Acids (RAP‐SNA)

RAP‐DNA conjugate was dissolved in DMSO and then dropwise added into PBS solution under stirring. The mixture was dialyzed against PBS solution overnight to generate the RAP‐SNA. 1% agarose gel electrophoresis was used to assess the assembling of RAP‐SNA. The diameter and morphology of RAP‐SNA were characterized by dynamic light scattering (Malvern Instruments, UK), transmission electron microscopy (TEM), and atomic force microscopy (AFM, Nanonavi E‐Sweep, Japan).

The as‐prepared RAP‐SNA could load functional segments by Watson‐Crick base pairing to prepare multifunctional SNA. For LOX‐1 siRNA and RAP coloading formulation preparation, the siRNA was designed with an overhang on the sense strand to hybridize with RAP‐SNA. The LOX‐1 siRNA sequences were designed according to previous studies.^[^ ^48^ ^]^ The RAP‐SNA and LOX‐1 siRNA were mixed in PBS buffer at room temperature with the ratio range from 1:0.05 to 1:0.25 in terms of nucleic acid. The successful assembly of LOX‐1/RAP‐SNA was characterized by 0.5% agarose gel electrophoresis using tris‐boric acid‐EDTA (TBE) as the running buffer. For imaging formulation preparation (Cy5.5/RAP‐SNA and Gd/RAP‐SNA), the Cy5.5‐polyA_15_‐Cy5.5 (or Gd‐DOTA‐polyA_15_) was mixed with RAP‐SNA in PBS buffer at a ratio of 0.2:1 in terms of DNA at room temperature and characterized by 0.5% agarose gel electrophoresis.

### ROS Stimulated Drug Release Assay

H_2_O_2_ was added to the RAP‐SNA solution and incubated at 37 ℃ (the final concentration of H_2_O_2_ 1 × 10^−3^ m). After different time intervals, the mixture solutions were centrifuged at 3000 rpm for 10 min and the drug concentration in the supernatant was determined by Ultraviolet spectrum (UV).

### In Vitro Cell Uptake of RAP‐SNA

RAW264.7 cells were seeded in a 24‐well plate at an intensity of 5 × 10^4^ cells per well and incubated in DMEM culture media overnight. Cy5.5/RAP‐SNA and Cy5.5‐polyA_15_‐ were added with the final Cy5.5 concentration of 500 × 10^−9^ m and incubated in Opti‐MEM culture media for different time intervals (0.5, 1, 2, 4, and 6 h). Cells were harvested for flow cytometry analysis.

### CLSM Imaging

RAW264.7 cells were seeded in a 24‐well plate with a clean coverslip at the bottom and incubated in DMEM overnight. Cy5.5/RAP‐SNA were added and incubated in Opti‐MEM for different time intervals (0.5, 2, and 4 h). Removed the medium and washed cells with PBS 3 times. The cells were immobilized with paraformaldehyde for 15 min, followed by rinsing the cells with PBS and staining the cell nucleus with Hoechst 33 342. Slides were mounted and visualized under the CLSM microscope (Leica TCS SP8 STED 3X, Germany).

### Cytotoxicity Assay

RAW264.7 cells were seeded in 96‐well plates and incubated in DMEM overnight. Thereafter, free RAP solution and RAP‐SNA were added with the RAP concentration range from 0.1 to 10 × 10^−6^ m. After incubated for 24 h, 20 µL MTT solution (5 mg mL^−1^) was added and incubated for another 4 h. Later on, removed the supernatant, followed by the addition of 150 µL DMSO to dissolve the formazan. The absorbance at 490 nm was recorded under a microplate reader (Synergy H4, Biotek, USA).

### Induction of Macrophages Autophagy by RAP‐SNA

RAW264.7 cells were seeded in 4 chamber glass‐bottom dishes and incubated in DMEM overnight, followed by refreshing culture medium and addition of RAP‐SNA with different RAP concentrations of 10, 25, and 50 × 10^−6^ m. After incubated for 24 h, the culture medium was removed and cells were rinsed with PBS 3 times, followed by the addition of paraformaldehyde to immobilize cells. Later on, cells were treated with Triton for 30 min, followed by adding the QuickBlock blocking buffer and incubated for 15 min. Thereafter, anti‐LC3B primary antibody was added and incubated at 4 ℃ for 24 h. Removed the antibody and rinsed cells with PBS, followed by the addition of Alexa Flour 555‐labeled secondary antibody. After incubation at room temperature for 2 h, the fluorescence signal was observed under the CLSM microscope.

### In Vitro LOX‐1 Downregulation and oxLDL Uptake

RAW264.7 cells were seeded in 24‐well plates and incubated in DMEM overnight. Thereafter, PBS, LOX‐1 siRNA, RAP‐SNA, LOX‐1/RAP‐SNA, and LOX‐1 siRNA transfected by lipofectamine 2000 (Lipo 2k/LOX‐1) were added and incubated in Opti‐MEM for 12 h, followed by refreshing the medium with DMEM and incubated for another 36 h. For LOX‐1 downregulation evaluation, proteins were extracted for western blot analysis. For oxLDL uptake, after the cells were treated with different formulations, DiI‐labeled oxLDL (Dil‐oxLDL) were added with the final concentration of 20 µg mL^−1^ and incubated for 5 h. Later on, cells were harvested and analyzed by flow cytometry (BD & LSR Fortessa, USA).

### Animals

All animal experiments were conducted under the laboratory animal’ care and use principle, and all operations were approved by the Shanghai Sixth People's Hospital Animal Experiment Welfare Ethics Committee (2020‐0604). All experiments involving animals were performed in accordance with the guidelines of the Institutional Animal Care and Use Committee (IACUC) of Shanghai Sixth People's Hospital (China). Male apolipoprotein E‐deficient (ApoE^−/−^) mice (6–8‐week‐old) were fed with a high‐cholesterol diet to establish the atherosclerotic model.

### Ex Vivo Plaque Targeting of RAP‐SNA and In Vivo MR Imaging

After feeding with a high‐cholesterol diet for 12 weeks, the Cy5.5 labeled RAP‐SNA (Cy5.5/RAP‐SNA) were intravenously injected into the ApoE^−/−^ mice at a dosage of 100 µg Cy5.5/kg mice. After different time intervals (1, 4, and 8 h), the mice were anaesthetized and went through heart perfusion to separate aorta for fluorescence imaging under the IVIS Spectrum (PerkinElmer). For in vivo MR imaging of the early‐stage atherosclerotic mouse, the mouse was fed with the high‐cholesterol diet for 5 weeks and then intravenously administrated with Gd/RAP‐SNA. The MRI scanning was performed before and 1 h postadministration of Gd/RAP‐SNA (4 µmol Gd kg^−1^ mouse) on an 11.7 T BioSpec MRI system equipped with a 12 cm gradient coil. The mouse was positioned in prone. The respiration and core temperature were monitored during the scan by the MR system compatible system. The scanning parameters were detailed as follows. T1‐RARE sequence; echo time, 7.5 ms; repetition time, 1000 ms; echo spacing, 7.5 ms.

### In Vivo Antiatherosclerosis Assay

6‐week old apoE^–/–^ mice were fed with a high‐cholesterol diet for 8 weeks. Thereafter, the atherosclerotic mice were intravenously injected with PBS, free RAP solution, RAP‐SNA, LOX‐1/RAP‐SNA, and LOX‐1 siRNA twice a week at a dosage of 4 mg RAP kg^−1^ mice and 0.5 mg siRNA kg^−1^ mice. After being treated for 7 times, the plasmas were collected for biochemical analysis. Thereafter, the mice were anaesthetized and went through heart perfusion with 30 mL PBS and 10 mL paraformaldehyde. The aortas connected with heart were harvested and preserved in paraformaldehyde. Thereafter, ≈80% ventricular part was discarded to embed the bottom part in paraffin. The 3 µm thick aortic root section was obtained after discarding ≈20 µm useless sections for Hematoxylin and Eosin (H&E) staining and immunohistochemical analysis. In addition, aortas were also separated for ORO staining. In addition, the main tissues (heart, liver, spleen, lung, and kidney) were reserved for H&E staining to evaluate the biosafety of the formulations.

### Statistical Analysis

All experiments were repeated at least three times, and the data were presented as the mean ± SD. The statistical significance was determined using the analysis of two‐tailed Student's *t*‐test, one‐way analysis of variance (ANOVA) with GraphPad Prism 6.0. Statistical significance was noted as follows: *, *P* < 0.05; **, *P* < 0.01; ***, *P* < 0.001; ****, *P* < 0.0001. Graph analysis was performed using GraphPad Prism 6.0 (GraphPad Software, USA).

## Conflict of Interest

The authors declare no conflict of interest.

## Supporting information

Supporting Information

## Data Availability

The data that support the findings of this study are available from the corresponding author upon reasonable request.
