# Supplementary material for: Plaque‐Targeted Rapamycin Spherical Nucleic Acids for Synergistic Atherosclerosis Treatment
Source: Adv Sci (Weinh). 2022 Mar 28;9(16):2105875. doi: 10.1002/advs.202105875 (PMC9165522; doi:10.1002/advs.202105875)
Supplement: Supplementary file 1 — Supporting Information [file ADVS-9-2105875-s001.pdf]

ADVANCED SCIENCE
Open Access

Supporting Information

for *Adv. Sci.*, DOI 10.1002/advs.202105875

Plaque-Targeted Rapamycin Spherical Nucleic Acids for Synergistic Atherosclerosis Treatment

Yuanyuan Guo, Jingcan Qin, Qianqian Zhao, Jiapei Yang, Xiaoer Wei, Yu Huang, Miao Xie, Chuan Zhang and Yuehua Li**

Supporting Information

Plaque-targeted rapamycin spherical nucleic acids for synergistic atherosclerosis treatment

Yuanyuan Guo, Jingcan Qin, Qianqian Zhao, Jiapei Yang, Xiaoer Wei, Yu Huang, Miao Xie, Chuan Zhang, and Yuehua Li**

Dr. Y. Guo, Dr. J. Qin, Q. Zhao, X. Wei, Dr. Y. Huang, and Prof. Y. Li

Department of Radiology, Shanghai Jiao Tong University Affiliated Sixth People's Hospital, Shanghai Jiao Tong University School of Medicine, 600 Yi Shan Road, Shanghai 200233, China.
E-mail: liyuehua77@sjtu.edu.cn (Yuehua Li)

J. Yang, M. Xie, and Prof. C. Zhang
School of Chemistry and Chemical Engineering, Frontiers Science Center for Transformative Molecules, Shanghai Jiao Tong University, 800 Dongchuan Road, Shanghai 200240, China.
E-mail: chuanzhang@sjtu.edu.cn (Chuan Zhang)

Table S1. The sequences of DNA and RNA strands used in this study.

Oligonucleotides	Oligonucleotide sequences (5'- 3')
polyT_{30}	TTTTTTTTTTTTTTTTTTTTT*T*T*T*T*T*T*T*T*T
polyA_{15}-Cy5.5	AAAAAAAAAAAAAAA-Cy5.5
polyA_{15}-NH_2	AAAAAAAAAAAAAAA-NH_2
Sense-LOX-1 siRNA (mice)	rArArArArArArArArArArArArArArArGrUrGrGrCrCrArGrUrUrArCr UrArCrArArArUdTdT
Antisense-LOX-1 siRNA (mice)	rArUrUrUrGrUrArGrUrArArCrUrGrGrCrCrArCdTdT
Sense-LOX-1 siRNA (human)	rArArArArArArArArArArArArArArArArGrGrArCrGrGrUrUrCrUrCrC rUrUrUrGrArUdTdT
Antisense-LOX-1 siRNA (human)	rArUrCrArArArGrGrArGrArArCrCrGrUrCrCrUdTdT

* represents for the phosphorothioate modification sites. The underlined segments could hybridize with RAP-SNA.

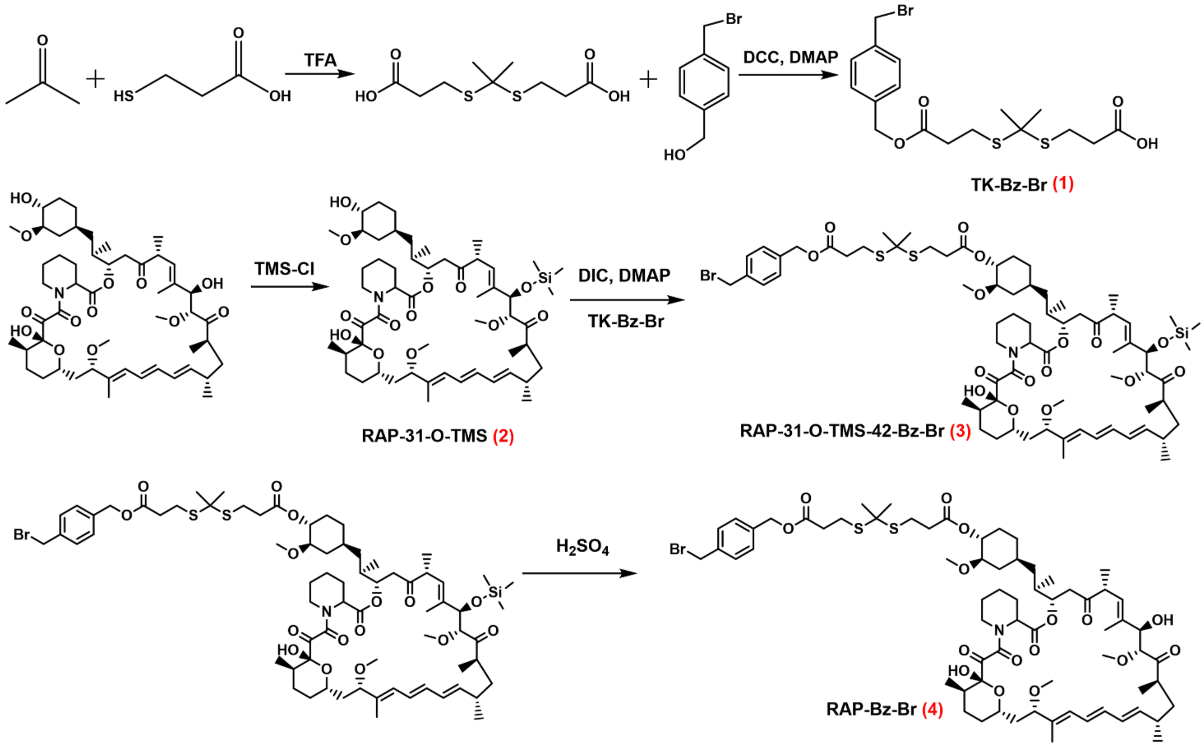

Figure S1. The chemical synthetic route of RAP-Bz-Br.

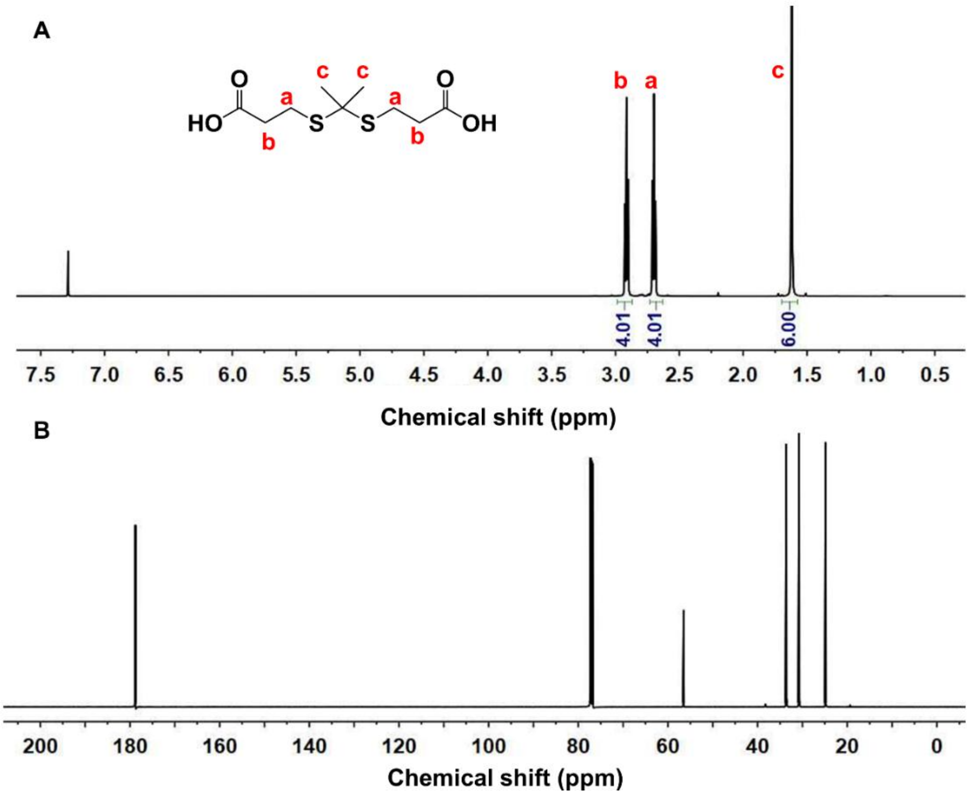

Figure S2. The ^{1}H-NMR (A) and ^{13}C-NMR (B) spectra of thioketal (TK).

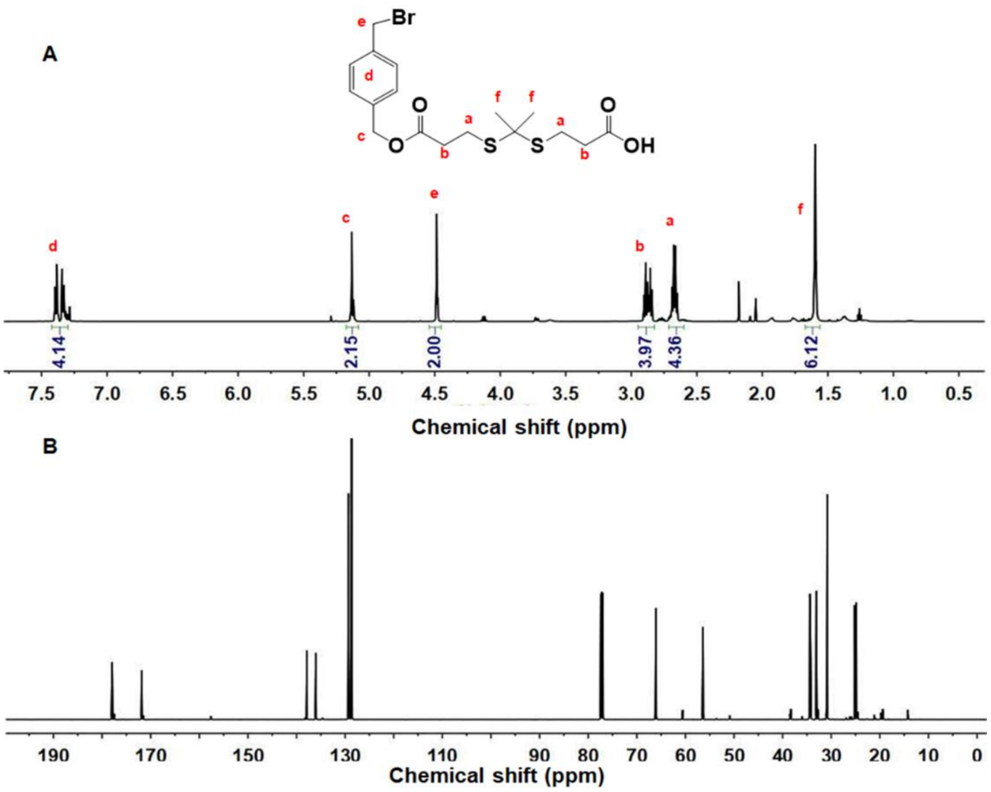

Figure S3. The ^{1}H-NMR (A) and ^{13}C-NMR (B) spectra of TK-Bz-Br (compound 1).

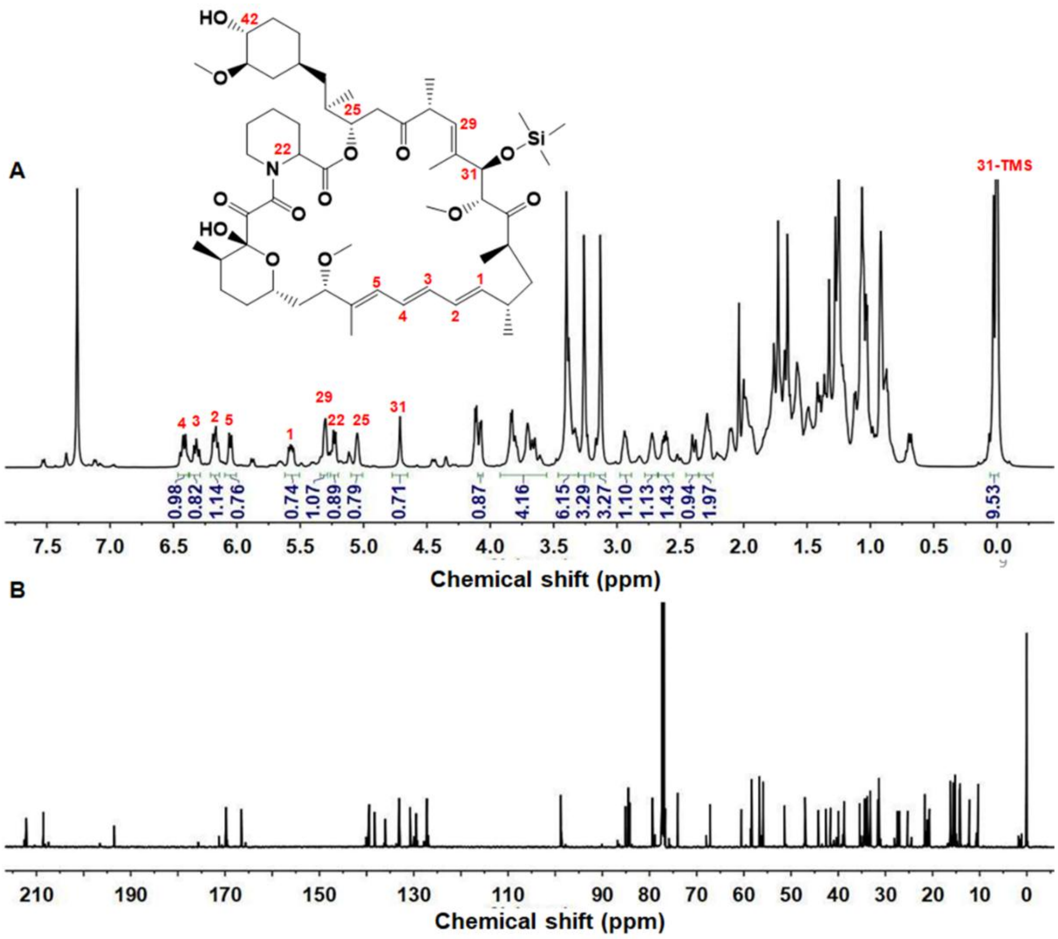

Figure S4. The ^{1}H-NMR (A) and ^{13}C-NMR (B) spectra of RAP-31-O-TMS (compound 2).

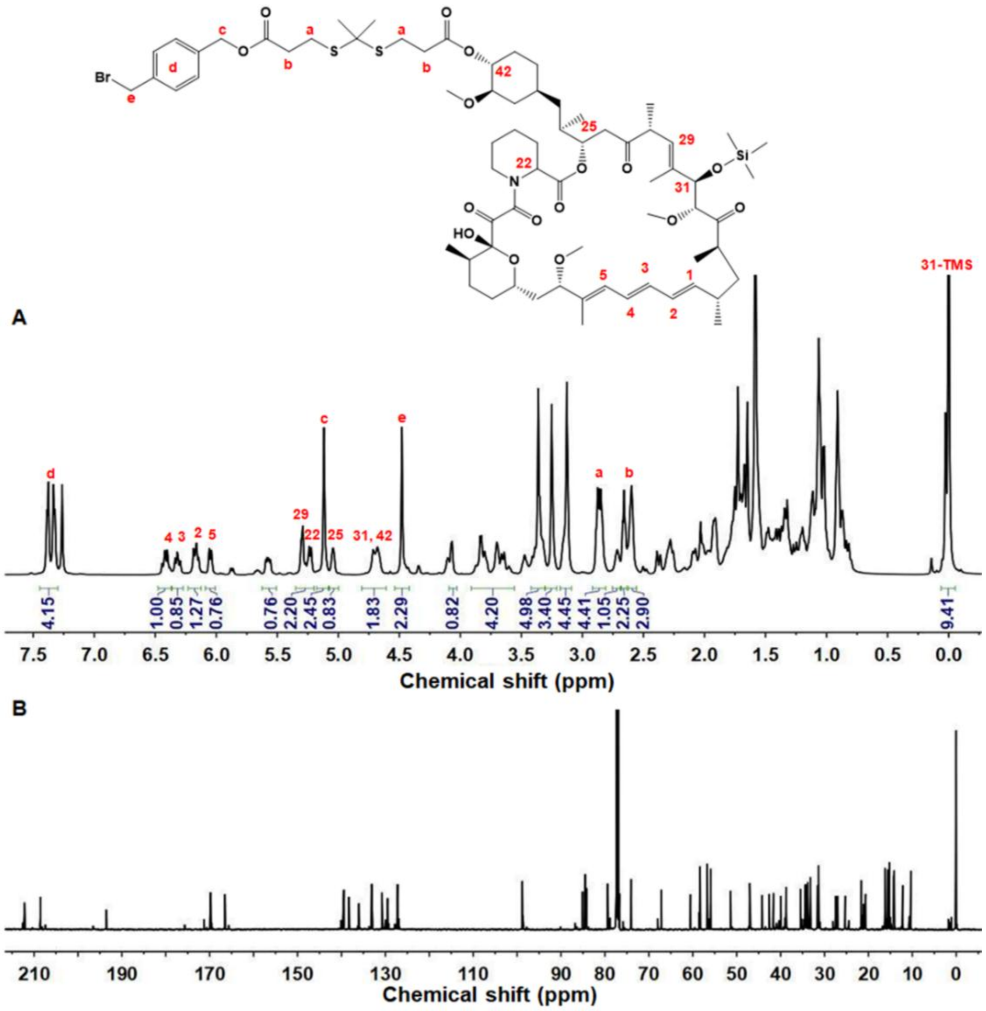

Figure S5. The ^{1}H-NMR (A) and ^{13}C-NMR (B) spectra of RAP-31-O-TMS-42-Bz-Br (compound 3).

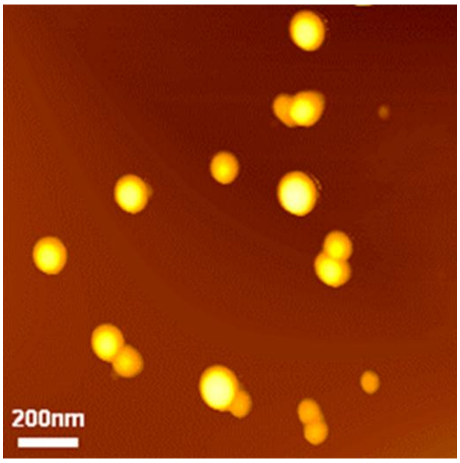

Figure S6. The morphology of RAP-SNA measured by atomic force microscope (AFM). Scale bar: 200 nm.

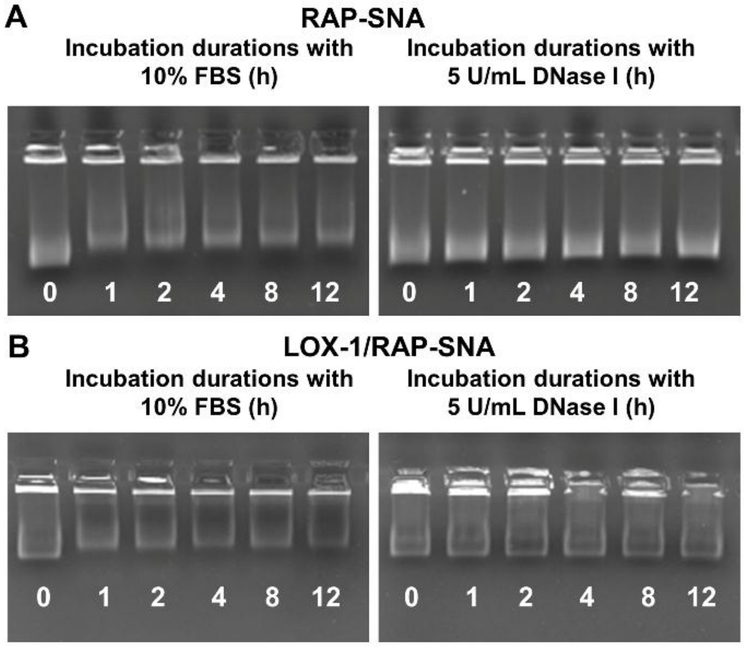

Figure S7. *In vitro* physiological stability of RAP-SNA and LOX-1/RAP-SNA. 0.5% agarose gel images of RAP-SNA (A) and LOX-1/RAP-SNA (B) after incubation with 10% fetal bovine serum (FBS)-containing buffer or 5 U/mL DNase I for different time intervals. The electrophoresis was performed under native condition.

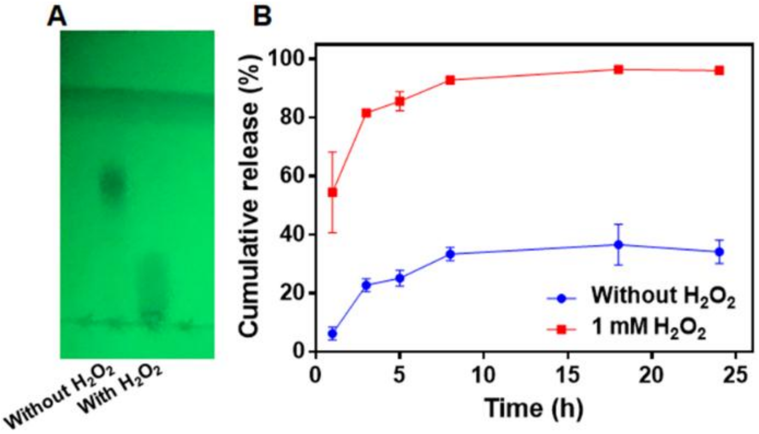

Figure S8. ROS-stimulated thioketal cleavage and drug release property of RAP-Bz-Br (A) and RAP-SNA (B).

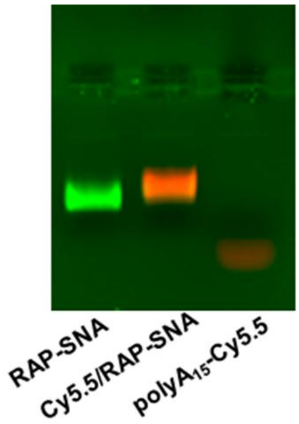

Figure S9. 0.5% agarose gel electrophoresis images of RAP-SNA, Cy5.5/RAP-SNA, and polyA$_{15}$-Cy5.5 using 1 × Tris-boric acid-EDTA/ Na^{+} (150 mM) solution as running buffer.

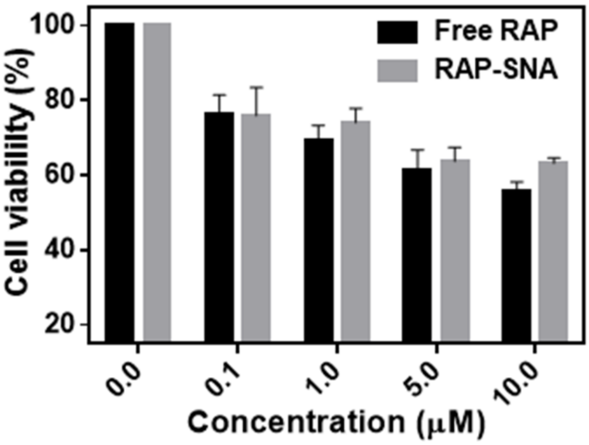

Figure S10. The cell viability of RAW264.7 cells after being treated with free RAP and RAP-SNA for 24 h.

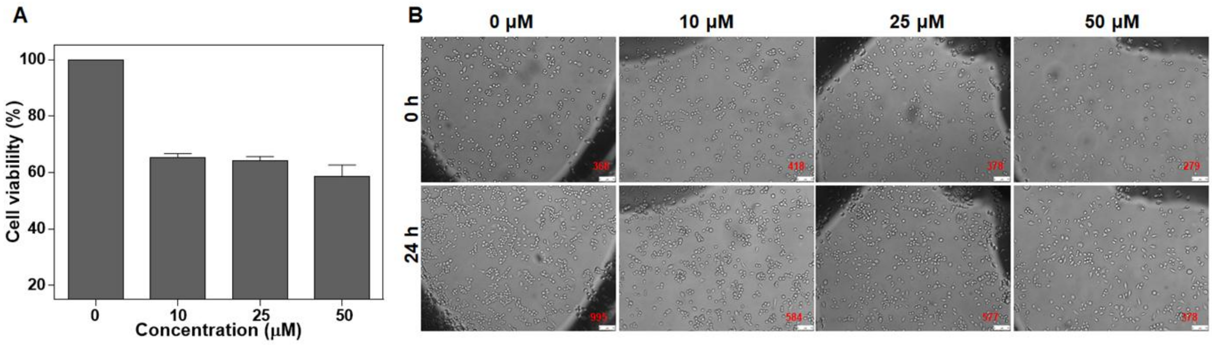

Figure S11. The cell viability (A) and microscopic images (B) of RAW264.7 cells after being treated with RAP-SNA for 24 h at high RAP concentrations. Red fond is the number for cell counts in each image. Scale bar, 75 μm.

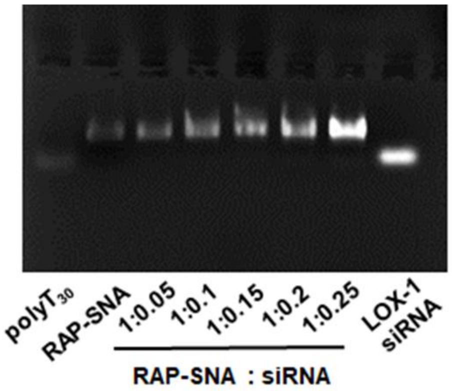

Figure S12. 0.5% agarose gel images of LOX-1/RAP-SNA with different RAP-SNA/siRNA ratios under native condition (Tris-boric acid-EDTA/ Na^+ (150 mM) buffer).

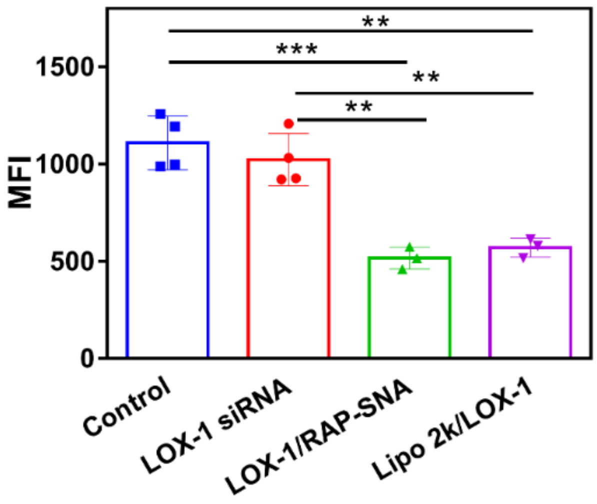

Figure S13. Flow cytometry analysis of the engulfing of DiI-oxLDL by THP-1-derived macrophages after treatment with LOX-1 siRNA, LOX-1/RAP-SNA, and Lipo 2k/LOX-1 siRNA. The humanized LOX-1 siRNA sequence is shown in Table S1. Cells were transfected with LOX-1 siRNA, LOX-1/RAP-SNA, and Lipo 2k/LOX-1 siRNA for 12 h, followed by refreshing the medium with DMEM and incubation for another 36 h. Then DiI-oxLDL was added and incubated for 3 h to perform the flow cytometry analysis. Statistical significance: **, $P < 0.01$; ***, $P < 0.001$.

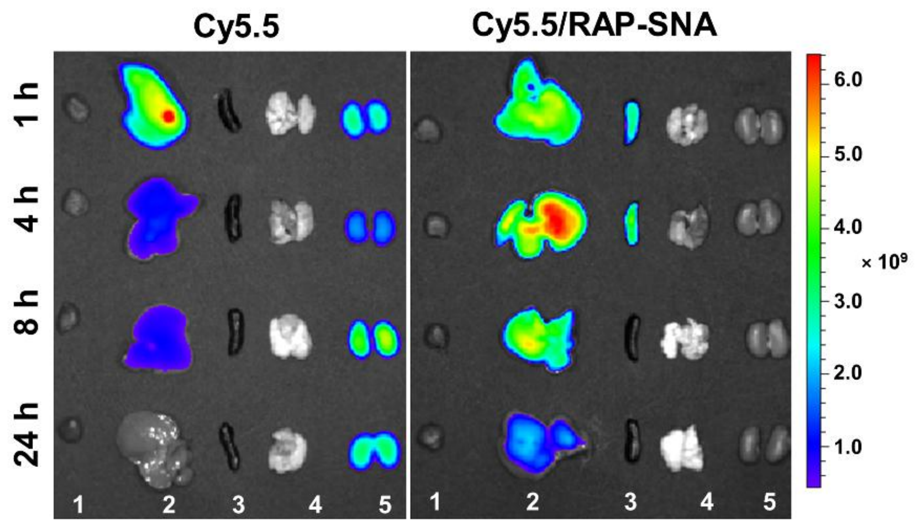

Figure S14. The bio-distribution results of free drug solution (Cy5.5) and SNA formulation (Cy5.5/RAP-SNA) in heart (1), liver (2), spleen (3), lung (4), and kidneys (5).

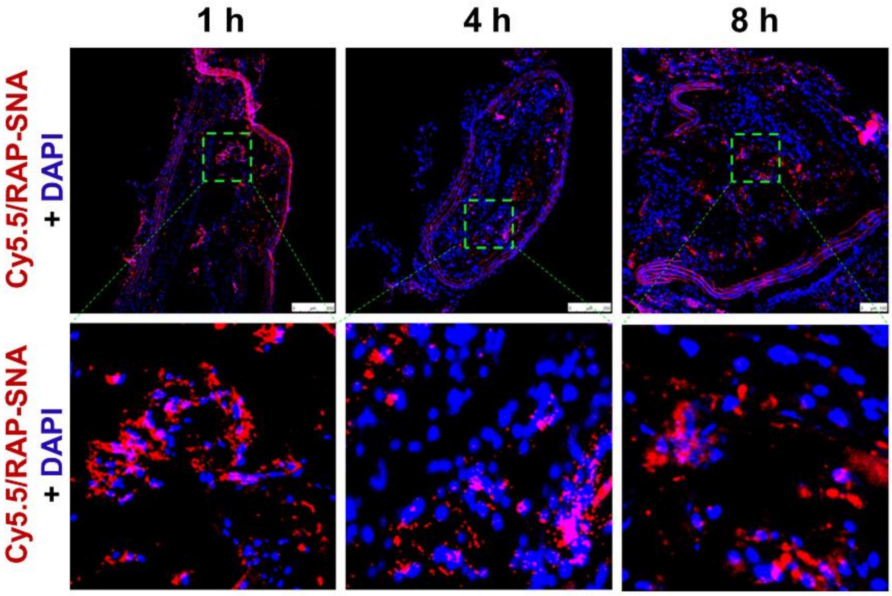

Figure S15. CLSM images of the frozen aorta cross-sections obtained from ApoE$^{-/-}$ mice after intravenously injecting Cy5.5/RAP-SNA to mice. Scale bar: 250 μm.

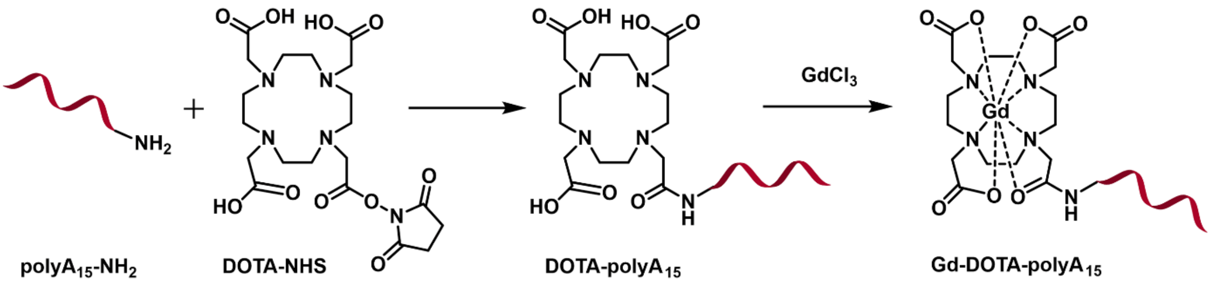

Figure S16. The synthetic route of DOTA-polyA_{15} and Gd-DOTA-polyA_{15}. DOTA-polyA_{15} was first synthesized by mixing polyA_{15}-NH_2 (1 eq.) with DOTA-NHS ester (100 eq.) in reacting solution (80% DMSO, 10% deionized water, and 10% 10 × HEPES, pH = 9) at 37 °C for 24 h. The DMSO and DOTA-NHS were removed by dialysis against water. The purified DOTA-polyA_{15} (1 eq.) was mixed with $GdCl_3$ (200 eq.) in HEPES buffer (pH = 5.5) and

incubated at 37 ℃ for 24 h. G25 desalination column was used to remove the small molecule $GdCl_3$. The collected solution was dried over to obtain the final purified Gd-DOTA-polyA$_{15}$.

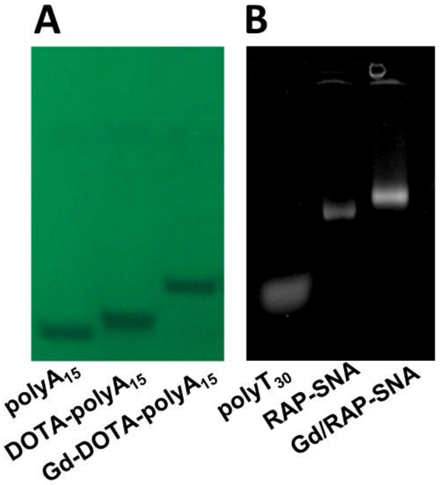

Figure S17. (A) 20% denaturing gel images of polyA$_{15}$, DOTA-polyA$_{15}$, and Gd-DOTA-polyA$_{15}$. (B) 0.5% agarose gel images of RAP-SNA and Gd/RAP-SNA.

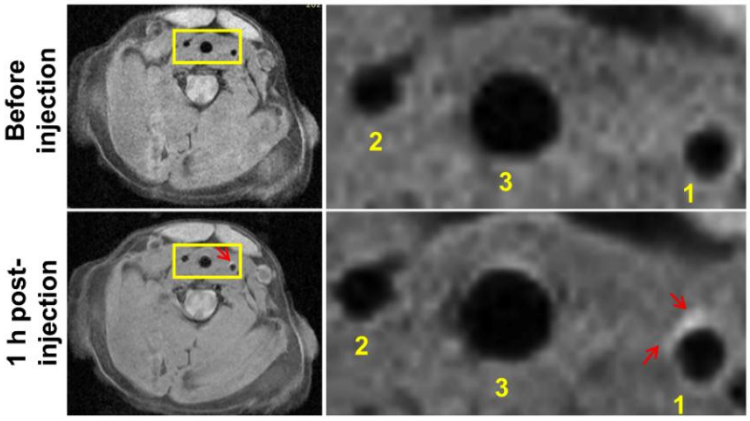

Figure S18. *In vivo* MR images of the carotid recorded before and 1 h post-injection of Gd/RAP-SNA. 1, left common carotid artery; 2, right common carotid artery; 3, trachea.

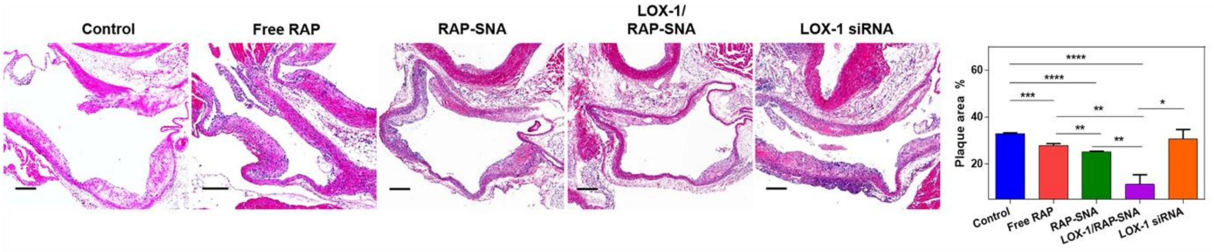

Figure S19. (A) H&E-stained images of the innominate arteries cross-section obtained from ApoE$^{-/-}$ mice after treatment with different formulations. (B) Statistical analysis of the plaque area based on the total area. Statistical significance: *, $P < 0.05$; **, $P < 0.01$; ***, $P < 0.001$; ****, $P < 0.0001$. Scale bar: 200 μm.

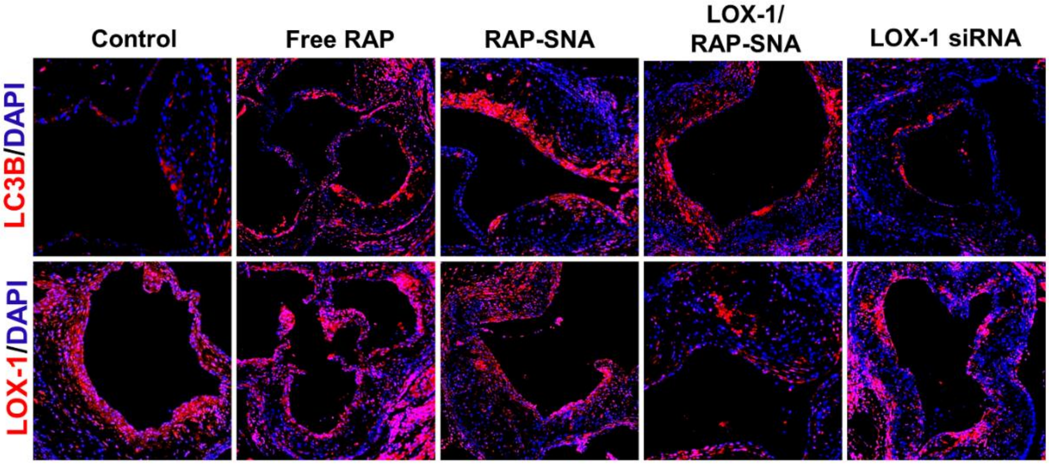

Figure S20. Anti-LC3B and anti-LOX-1 antibodies-stained images of aortas cross-sections to evaluate the autophagy and LOX-1 expression in plaques after different treatments.
